# Supplementary material for: Development of phage-containing hydrogel for treating Enterococcus faecalis-infected wounds
Source: PLoS One. 2024 Oct 28;19(10):e0312469. doi: 10.1371/journal.pone.0312469 (PMC11515978; doi:10.1371/journal.pone.0312469)
Supplement: Attachment — Submitted filename: PLOS ONE comments.docx [file pone.0312469.s001.docx]

In the manuscript titled “Development of Phage-containing Hydrogel for Treating Enterococcus faecalis Infected Wounds”, the authors reported on a novel hydrogel incorporating SAM-E.f 12 and its application in treating wounds infected with Enterococcus faecalis. However, the present quality of this manuscript cannot reach the acceptance level for publication in *PLOS ONE*. There are some major comments and questions for the revision of the manuscript.

Comment 1: In Figure 10, the authors lack quantitative data on the wound healing rate in mice, relying solely on photographs of wound healing at different time points, which is not sufficiently rigorous.

Comment 2: The potential for bacteria to develop resistance to phages over time is not addressed. This is a significant concern in phage therapy and should be discussed in the context of the study's findings.

Comment 3: While the study discusses the stability of the hydrogel in vitro, there is limited information on its stability and performance under various physiological conditions, such as different pH levels.

Comment 4: Please check that the abbreviation is explained at the first mention of each section to facilitate the manuscript reading.

Comment 5: Authors should carefully check the grammar errors, formation, and spelling in the manuscript. In general, the manuscript should also be improved with editing by a native English speaker for grammatical and language mistakes.

Comment 6: A good excellent academic paper should not only show the experimental results, but also give an in-depth discussion on these results. The author should pay attention to it. Some related studies (such as *Exploration* 10.1002/EXP.20210083、10.1002/EXP.20230066、10.1002/EXP.20220173、*ACS Appl Mater Interfaces*10.1021/acsami.3c16061、*NPG Asia Materials* 10.1038/s41427-022-00444-x) can be cited and discussed to improve the discussion of this work.
